# TarPan: an easily adaptable targeted sequencing panel viewer for research and clinical use

**DOI:** 10.1186/s12859-020-3477-y

**Published:** 2020-04-15

**Authors:** Cody Ashby, Michael Rutherford, Michael A. Bauer, Erich A. Peterson, Yan Wang, Eileen M. Boyle, Christopher P. Wardell, Brian A. Walker

**Affiliations:** 1 Department of Biomedical Informatics, University of Arkansas for Medical Sciences, Little Rock, AR USA; 2 Cancer Institute: Myeloma Center, University of Arkansas for Medical Sciences, Little Rock, AR USA; 3 Division of Hematology Oncology, Indiana University, Indianapolis, IN USA

**Keywords:** Cancer, Visualization, Somatic mutations, Structural variants, Copy number

## Abstract

**Background:**

The study of cancer genomics continually matures as the number of patient samples sequenced increases. As more data is generated, oncogenic drivers for specific cancer types are discovered along with their associated risks. This in turn leads to potential treatment strategies that pave the way to precision medicine. However, significant financial and analytical barriers make it infeasible to sequence the entire genome of every patient. In contrast, targeted sequencing panels give reliable information on relevant portions of the genome at a fiscally responsible cost. Therefore, we have created the Targeted Panel (TarPan) Viewer, a software tool, to investigate this type of data.

**Results:**

TarPan Viewer helps investigators understand data from targeted sequencing data by displaying the information through a web browser interface. Through this interface, investigators can easily observe copy number changes, mutations, and structural events in cancer samples. The viewer runs in R Shiny with a robust SQLite backend and its input is generated from bioinformatic algorithms reliably described in the literature. Here we show the results from using TarPan Viewer on publicly available follicular lymphoma, breast cancer, and multiple myeloma data. In addition, we have tested and utilized the viewer internally, and this data has been used in high-impact peer-reviewed publications.

**Conclusions:**

We have designed a flexible, simple to setup viewer that is easily adaptable to any type of cancer targeted sequencing, and has already proven its use in a research laboratory environment. Further, we believe with deeper sequencing and/or more targeted application it could be of use in the clinic in conjunction with an appropriate targeted sequencing panel as a cost-effective diagnostic test, especially in cancers such as acute leukemia or diffuse large B-cell lymphoma that require rapid interventions.

## Background

The field of cancer genomics has, and continues, to rapidly advance. Large-scale genomic studies have revealed a host of information about a wide variety of cancer types that have led to genetic-driven targeted therapy. However, despite these advancements, due to cost it currently remains infeasible to run whole genome or exome sequencing for every patient. To that end, genomic researchers and clinicians have employed the use of targeted sequencing panels that reveal driver mutations, copy number and structural events relevant to their specific cancer of interest that may be used for diagnostic, prognostic, and theranostic purposes.

Tools such as Integrative Genome Viewer [1] and the Integrated Genome Browser [2] have been designed to view large-scale genomic data and are well-suited for that purpose. However, while it is certainly possible, it is arduous to analyze targeted sequencing samples using conventional tools developed with whole-genome or whole-exome data in mind. For example, to analyze somatic events such as mutations, copy number changes, and structural variants would require the user to load multiple tracks and bam files and manually move the browser to each region of interest. Further, the hardware requirements to load all this data can be burdensome. In response to this problem, tools have begun to appear to aid researchers in these types of analysis such as CNSpecter [3], which is a browser tool that aims to facilitate the inspection of somatic copy number changes. Still, there remains the need for a viewer that can allow a researcher to easily inspect mutations, copy number changes, and structural variants in one application. In response to this problem we have developed the **Tar**geted **Pan**el (TarPan) Viewer.

### Implementation

#### Tool development

**Fig. 1:**
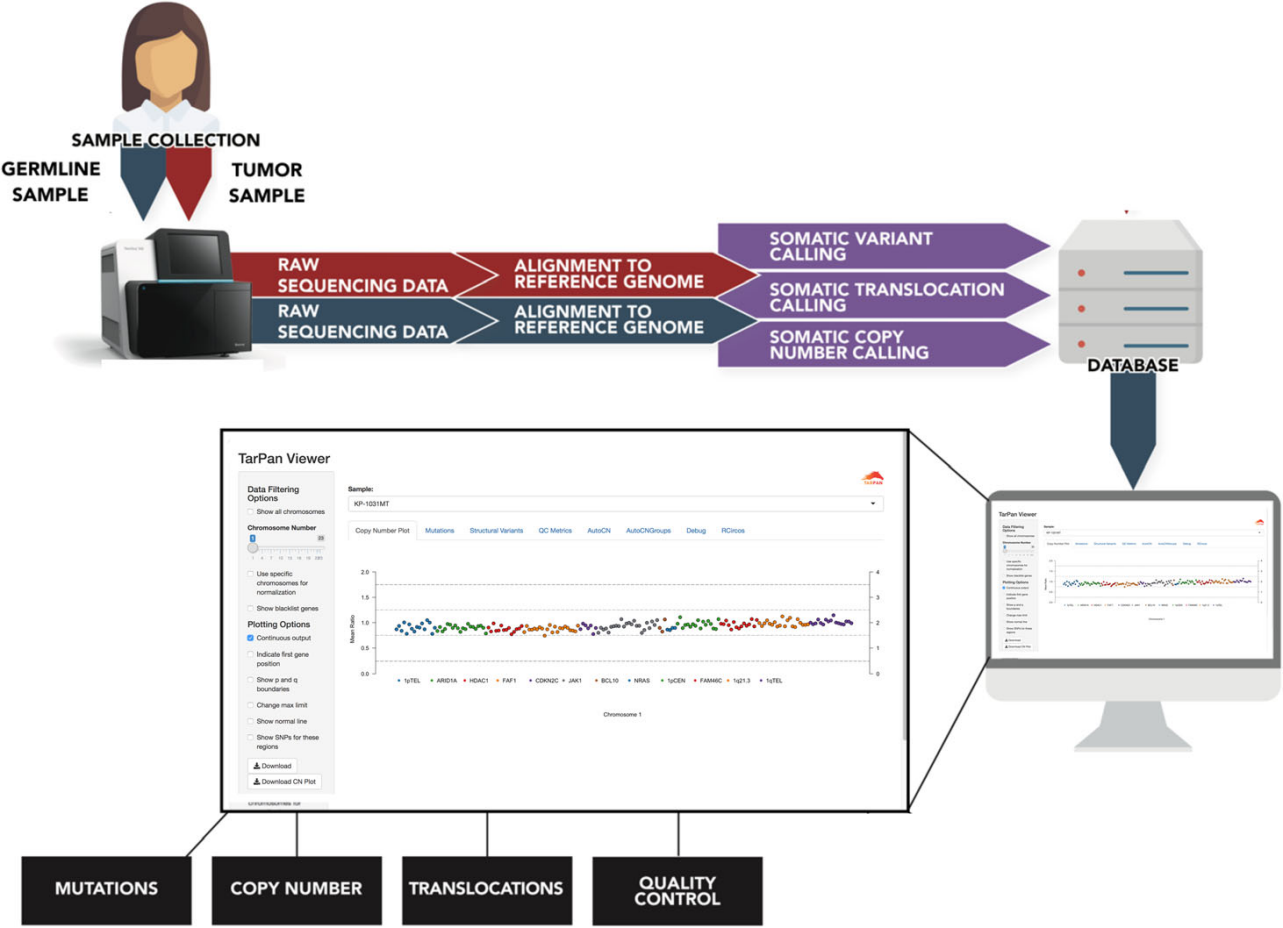
Overall flow chart of TarPan Viewer. Diagram of the overall process for importing samples into TarPan Viewer. Samples are aligned to a reference genome, processed through copy number, structural variant and mutation calling and imported into an SQLite database file. That database file is read by TarPan Viewer and displayed in an R Shiny user interface

TarPan Viewer is written in R Shiny and utilizes argument-driven python scripts to manage targeted panel sequencing data within SQLite databases. The complete design diagram for the tool is shown in Fig. 1. These scripts handle all database functions including creating, updating, and deleting databases and the targeted panel sequencing entries within them.

R Shiny was utilized for its simplicity and ability to harness the power of R’s vast libraries and rich visualization capabilities. Most important is its ability to scale, ensuring smooth access to accommodate a considerable number of users and sizable datasets. R Shiny along with SQLite met our need for cross-platform portability and reliability, no matter the file size.

#### Installation

TarPan is available (with example data) on GitHub (https://github.com/tcashby/tarpan) and can be installed on all platforms capable of running R Shiny including Windows, macOS, and Linux. Besides R Shiny, all R dependencies must be installed using the included script. Finally, a Python environment with the pandas module [4] is required to use the import scripts. TarPan may either be run in local or server mode depending on the version of R Shiny installed by the user.

#### Data preparation

TarPan accesses an SQLite database file generated from user provided input and is converted to SQLite tables by a series of custom python scripts. A single database (see Supplemental Figure 1 for database schema) file containing example data is included in the repository. Users may view and interact with the example database, or their specific databases using a standard SQLite database viewer.

The first step is creation of a non-populated database that contains the tables and required information that applies to all samples imported into this specific database. The required data to create an initial database are the output name of the database, the reference genome used (hg19 and hg38 are supported), the pipeline version used, a Browser Extensible Data (BED) file containing the targeted regions of the panel, and a BED file that contains the gene group annotations. All samples that were processed using the same required data may be grouped within the same database. Finally, the user must specify the name of the database in the configuration (config.yml) file for it to appear in TarPan Viewer.

The scripts are designed to take as input standardized formats such as BED and Variant Call Format (VCF). This allows the inclusion of a wide variety of algorithms and tools dependent on user preference. Non-standard formats must be converted according to the instructions specified in the installation document. After conversion of user data to SQLite tables, they are accessed by the RSQLite R package by TarPan and visualized using in a Shiny compatible browser of the user’s choice.

#### Testing pipeline software

While TarPan is designed to accept common data formats (e.g. BED and VCF files) from a variety of tools, initial testing was performed with the following tools:

- **Copy number:** depths provided by CNVKit (ver 0.9.5) [5]
- **Somatic variants:** provided by Strelka2 (ver. 2.9.10) [6]
- **Structural variants**: provided by Manta (ver. 1.5.0) [7]
- **Annotations**: provided by Variant Effect Predictor (ver. 95.3) [8]

## Results and discussion

### TarPan user interface

#### Overview

**Fig. 2:**
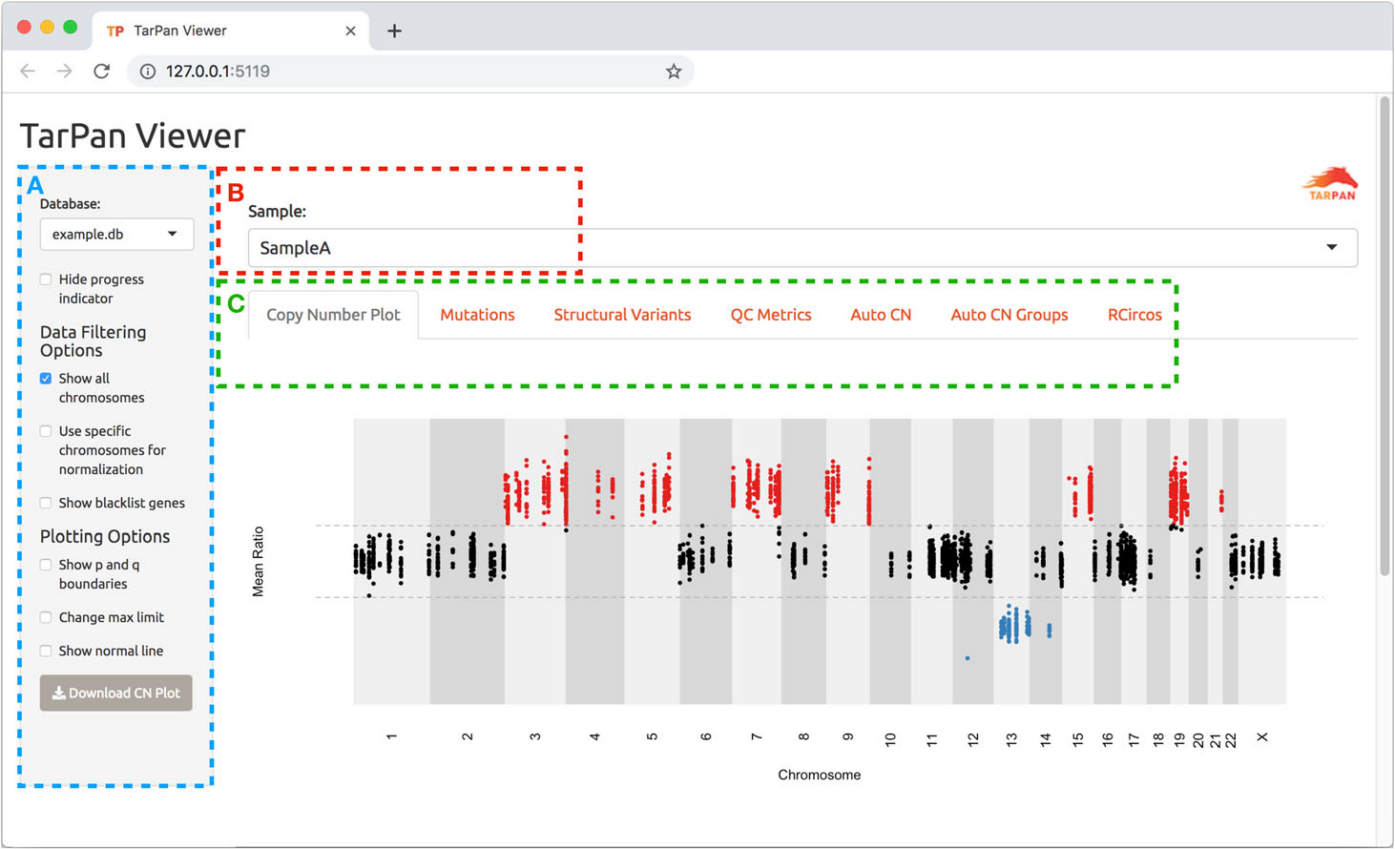
TarPan Viewer user interface. A screen capture of TarPan Viewer. The region in blue (**a**) shows the user filtering and option selection. The region in red (**b**) shows the current sample and allows for sample selection. The region in green (**c**) shows the current view selected and allows for view selection, in this example the copy number plot view is shown

The TarPan user interface is presented via any web browser. Options for data filtering and view specific options are provided on the left in a panel and the user may switch between views by clicking on tabs at the top. An image showing the interface for TarPan viewer is provided in Fig. 2.

#### Database selection

If the user has more than one database containing samples, they may select the current one by the dropdown in the selection pane.

#### Data filtering

TarPan offers the user the ability to filter based on all data available or on specific chromosomes. This filtering applies to all views in TarPan, including the copy number, mutation and structural variant views. Further, if a blacklist BED file is provided to the tool, those regions are hidden by default. If the user wishes to see these blacklist regions they may select the “Show blacklist genes” option.

#### Copy number calculation and visualization

By default, TarPan will use the ratio values provided by the tool used for depth comparison. If these values are not provided, the tool will calculate a normalized ratio based on chromosomes 1–22. Further adjustments may be made by the user at the whole chromosome level by selecting the “Use specific chromosome for normalization” option and selecting which chromosomes to use. This is especially useful for samples with highly aberrant chromosomes.

Copy number ratio is calculated by the following formula:

$$ R=\frac{T/{T}_{nf}}{N/{N}_{nf}} $$

Where *R* is the copy number ratio, *T* is the read depth for a given interval in the tumor sample, *N* is the read depth for the corresponding interval in the normal sample, *T*_*nf*_ is the tumor normalization factor generated by taking the mean of all given interval depths in the tumor sample and *N*_*nf*_ is the normal normalization factor generated by taking the mean of all given interval depths in the normal sample.

A user may select any combination of chromosomes using checkboxes for manual correction of normalization. Whenever manual correction is employed, only intervals located on the chromosomes that the user selects are used for the normalization. This is useful in cases where certain chromosomes seldom have any copy number aberrations (CNAs).

**Fig. 3:**
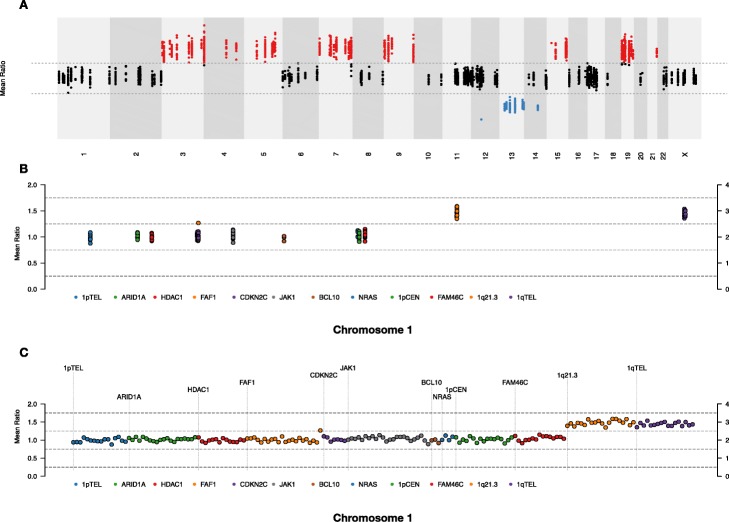
Copy number view options. Three different options for viewing copy number in TarPan Viewer. **a** The whole genome view, targeted capture intervals are shown as dots and colored based on whether they are gained (red), neutral (black) or deleted (blue). **b** Individual chromosomes view (chromosome 1 shown). Intervals are colored based on which gene region they are in and positioned according to their position in the chromosome. **c** Individual chromosome view with the continuous option selected. Intervals are colored based on which gene region they are in and positioned equidistantly from each other

In addition, there are three views provided for copy number inspection by TarPan. The first is the Whole Genome View in which all intervals on chromosomes are visible and positioned according to their genomic position. The second is Chromosome View in which all intervals on a single user selected chromosome are visible and positioned according to their genomic position. The third is Chromosome Continuous View in which all intervals on a single user selected chromosome are visible and positioned equidistantly in order to more easily view patterns for specific genes. An example of all three views are shown in Fig. 3.

Finally, the user may see visible SNP differences by clicking the “Show SNPs for these regions” option. This shows a plot with the SNPs that were seen as heterozygous in the normal and how they compare in the tumor sample. This data provides the user with the ability to identify where there is loss of heterozygosity in regions where the copy number is more than 1 as well as more confidence in regions of deletion.

#### Structural variant visualization

Structural variants may be inspected by selecting the structural variants tab. This provides a view of all structural variants that have PASS or “.” in the FILTER column of the VCF file. Optionally the user may choose to view all structural variants detected by the variant caller by selecting the “Show SVs that failed filter” checkbox. Variants are presented in a table, which is searchable and sortable.

#### Mutation visualization

Mutations may be inspected by selecting the mutation tab. This provides a view of all mutations that had PASS or “.” in the FILTER column of the VCF file. Optionally the user may choose to view all mutations detected by the variant caller by selecting “Show mutations that failed filter”. The algorithm detects annotations produced by the variant effect predictor (VEP) and extracts information such as gene symbol, consequence, amino acid protein code, nucleotide codon code, etc. if available. Otherwise, the raw VCF is displayed. Mutations are presented in a table, which is searchable and sortable.

#### Auto CN and auto CN groups

Although a strength of TarPan is in the ability of the user to easily manually inspect Copy Number (CN) regions, the user may click the Auto CN and Auto CN Groups tab to view a table containing calls made by the viewer itself. Auto CN shows the user a list of all normalized depth ratios for each interval in the targeted region bed file provided. Auto CN Groups shows the user a list of copy number calls for the entire group for each interval in the gene group BED file provided.

#### Circos visualization

A Circos-style visualization is provided by the RCircos package [9]. Structural variants, copy number and mutations appear on this Circos plot. Users may choose to hide/show any of these features by selecting the appropriate option. In addition, in the case of translocations users may choose to show the partner chromosome by selecting the “Show Inter-chromosomal SVs” option.

### TarPan case studies

#### Visualization of multiple myeloma data using TarPan

**Fig. 4:**
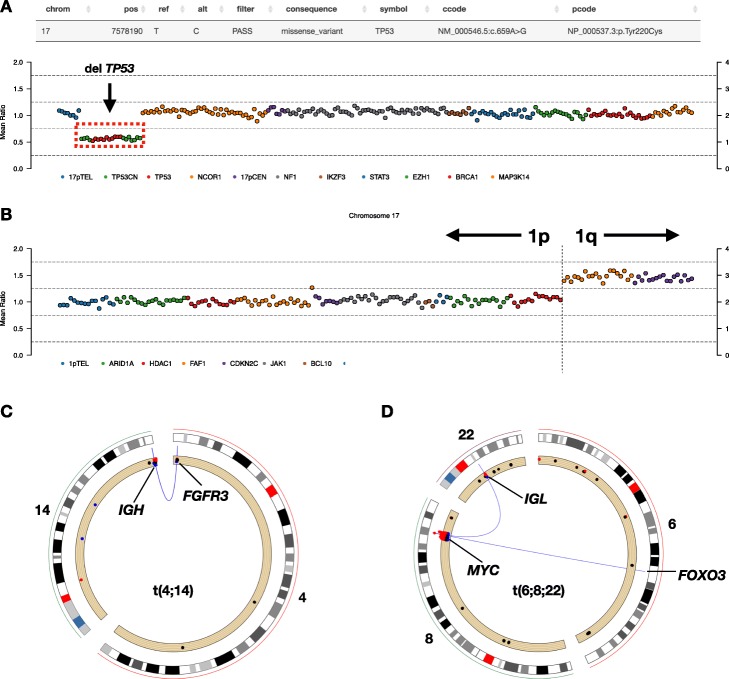
Multiple Myeloma examples. Several examples of features identified by TarPan viewer on multiple myeloma samples. **a** bi-allelic inactivation of *TP53* (mutation + copy number deletion)*.* **b** a gain on chromosome 1q. **c** A t (4;14) translocation event (a poor prognosis indicator). **d** A complex rearrangement in the proto-oncogene *MYC* which also involves *FOXO3* and *IGL,* t (6;8;22)

We currently have used TarPan for the visualization and analysis of 100 multiple myeloma samples which are available under European Genome Archive (EGA) accession number EGAS00001002859. In addition, there have been several peer reviewed publications [10–13] where TarPan was used for screening, validation, or analysis. Examples showing important multiple myeloma events in TarPan are shown such as bi-allelic inactivation of *TP53* (Fig. 4a), gain of 1q (Fig. 4b), translocations to the Ig regions (Fig. 4c) and complex structural events involving the proto-oncogene *MYC* (Fig. 4d)*.* Data from two publicly available multiple myeloma samples are provided as example data in the GitHub repository.

#### Visualization of follicular lymphoma data using TarPan

**Fig. 5:**
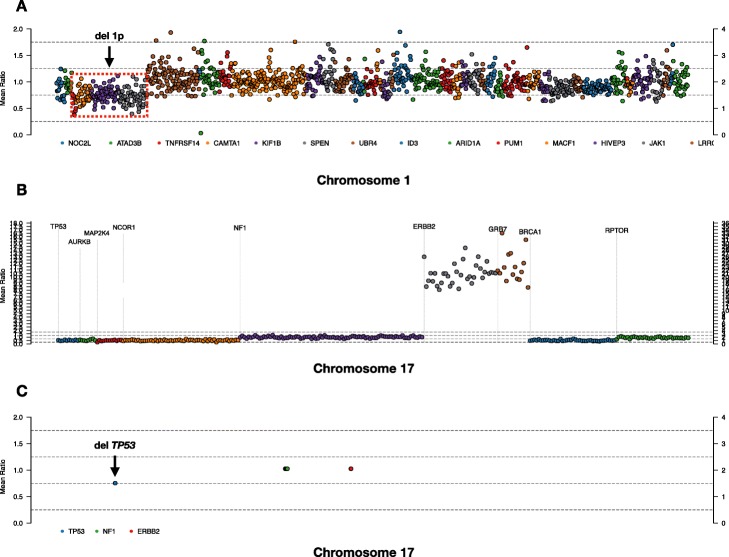
Follicular Lymphoma, breast and lung cancer examples. Examples of features identified by TarPan viewer on breast and lung cancer samples. **a** follicular lymphoma sample having a potential deletion in 1p impacting genes *TNFRSF14*, *CAMTA1*, *KIF1B*, *SPEN* (**b**) breast cancer sample having a massive focal amplification event on 17q impacting *ERBB2* (*HER2*) and *GRB7*. **c** lung cancer sample having a bi-allelic inactivation of *TP53* (mutation + copy number deletion)

Whole exome sequencing data was downloaded for patient TCRBOA7 from the open-access Texas Cancer Research Biobank [14]. This patient sample was obtained from a white male aged 61–70 years old, diagnosed with B-cell follicular lymphoma (FL). To analyze this data in TarPan we created a pseudo-targeted panel by limiting the exome to 490 genes used by the FL custom panel reported by Bouska et al. [15] plus two genes reported as mutated in TCRBOA7 [14]. We then ran the sample through the best practices pipeline described above and visualized the result in TarPan. We observed all previously reported mutations (*NFE2L3*, *KMT2D*, *CREBBP*) [5]. We also observed a deletion event in 1p (shown in Fig. 5a) that affected four genes on the pseudo-targeted panel which was also shown by ichorCNA [16] on WGS data (shown in Supplemental Figure 3).

#### Visualization of breast cancer cell line using TarPan

Whole genome sequencing data was obtained for the publicly available breast cancer cell line HCC2218C from Illumina Basespace with matching baseline sample B-lymphoblastoid cell line HCC2218BL. To analyze this data with TarPan we created a pseudo-targeted panel based on 79 genes referenced in [17]. We found one mutation in the gene *MIEN1.* We compared the copy number data from the panel to the results of SNP array data [18]. We observed similar features including a massive amplification on chromosome 17 (Shown in Fig. 5b) that impacted genes *ERBB2* (*HER2*) and *GRB7*, both of which can be seen in the previously reported data [18] and both of which have been reported as overexpressed or amplified in breast cancer [19, 20].

#### Visualization of lung cancer data using TarPan

We obtained BAM files for a formalin-fixed paraffin-embedded lung cancer tissue specimen along with its paired normal from the Johann Lab at the UAMS Cancer Institute. The panel used for these samples contained 72 genes of interest in lung cancer. The germline sample was sequenced to a mean depth of 1322 and the tumor was sequenced to a mean depth of 7365. We identified 8 mutations in 7 genes: *LRP1B*, *NFE2L2*, *GRM8*, *NTRK2*, *TP53*, *MUC16*, and *SMARCA4.* As this panel was strictly a mutation panel and did not include copy number regions we used low-depth WGS with ichorCNA (shown in Supplemental Figure 3) to calculate the depth ratio for targeted genes. This sample included a deletion in in 17p, which in conjunction with the mutation suggests bi-allelic inactivation of *TP53* (shown in Fig. 5c).

### TarPan use cases

#### Targeted panel visualization

The primary intended use for TarPan is to visualize data generated from targeted panel sequencing experiments. These can be mutation panels, copy number panels, structural variant panels or any combination thereof. Two of the case studies above show data generated from this use case. The multiple myeloma case study shows the results from a mutation + copy number + translocation targeted panel and the lung cancer case study shows the results from a mutation targeted panel. However, the user may also use other sequencing modalities to refine the results by providing the input to TarPan. An example of this is shown with the lung cancer sample by using low depth WGS to capture copy number depth ratios for genes of interest.

#### Whole-exome or genome visualization

While the main focus of TarPan is in the visualization of targeted panel sequencing data, as shown above in the follicular lymphoma and breast cancer case studies there are also applications in the field of conventional whole genome and exome sequencing. For example, if a researcher is interested in a smaller subset of genes to answer a specific question, they can easily reduce the data to a more manageable size and use TarPan to visualize the result. A similar use case is in the rapid prototyping of targeted sequencing panels and visualizing the type of information they might provide. For example, a user could limit a more comprehensive exome or genome sample to regions of interest and observe an approximation of what the data would look like on a targeted panel.

#### Database queries

As TarPan Viewer uses a simple SQLite database file as input, it is also possible for researchers familiar with SQL to easily batch query the entire database. For example, the user could query the copy number or mutation status of a captured interval across all samples.

### TarPan future development

There are several potential future developments planned for TarPan. The first is the ability to process and import tumor-only samples, which would be especially useful whenever a paired normal is unavailable such as in some cell line data. The second is a user annotation view that would allow users to manually annotate features of interest. The third is the inclusion of genomic data of different species, such as mouse. Currently TarPan only works on human genomic data (hg19 and hg38). Allowing custom genomic data would open up many new use cases for the viewer. Finally, we think the tool could easily be adapted to potentially aid in cost-effective clinical diagnostic tests using targeted sequencing by automatically generating reports or summaries (e.g. cancer subtypes or risk scores), which could be manually validated by a trained scientist.

## Conclusion

TarPan Viewer was developed to aid researchers in visual inspection and exploration of targeted panel sequencing data. Conventional genomic viewers designed for whole-genome or exome data are ill-suited to visualizing data from targeted panel sequencing data. In contrast, TarPan provides easily understood copy number, mutation and structural variant plotting and searchable tables of information relevant to a sample of interest. Its fast and responsive interface runs in a standard browser on any operating system capable of running R Shiny. The tool and scripts are easy to install, requiring only basic knowledge of R and Python, and step-by-step instructions are provided. The included example datasets demonstrate common use cases and highlight TarPan’s key features. The viewer has been well-utilized internally by trained bioinformaticians and cancer biologists to annotate samples. We believe that the described use cases and features illustrate its distinct usefulness. TarPan fills a clear need in the greater scientific community, helps advance cancer research, and is a step closer to the realization of personalized medicine.

## Supplementary information

**Additional file 1.**

**Additional file 2.**

**Additional file 3.**

## Data Availability

The source code and installation instructions for TarPan can be found at: https://github.com/tcashby/tarpan. ·Project name: TarPan Viewer ·Project home page: https://github.com/tcashby/tarpan ·Operating system(s): Platform Independent ·Programming language(s): Python, R ·Other requirements: Python 3, R 3.5 or higher ·License: GPL v3 ·Any restriction to use by non-academics: non-applicable

## Abbreviations

BED: Browser Extensible Data (file type)
CN: Copy Number
CNA: Copy Number Aberrations
CNV: Copy Number Variant
EGA: European Genome Archive
FL: Follicular Lymphoma
Ig: Immunoglobulin
SNP: Single Nucleotide Polymorphism
SQL: Structured Query Language
SV: Structural Variant
TarPan Viewer: Targeted Panel Viewer
UAMS: University of Arkansas for Medical Sciences
VCF: Variant Call Format (file type)
VEP: Variant Effect Predictor
WES: Whole-Exome Sequencing
WGS: Whole-Genome Sequencing
YML: YAML Ain’t Markup Language (file type)
