# Supplementary material for: TarPan: an easily adaptable targeted sequencing panel viewer for research and clinical use
Source: BMC Bioinformatics. 2020 Apr 15;21:144. doi: 10.1186/s12859-020-3477-y (PMC7158102; doi:10.1186/s12859-020-3477-y)
Supplement: Supplementary file 1 — Additional file 1. [file 12859_2020_3477_MOESM1_ESM.pdf]

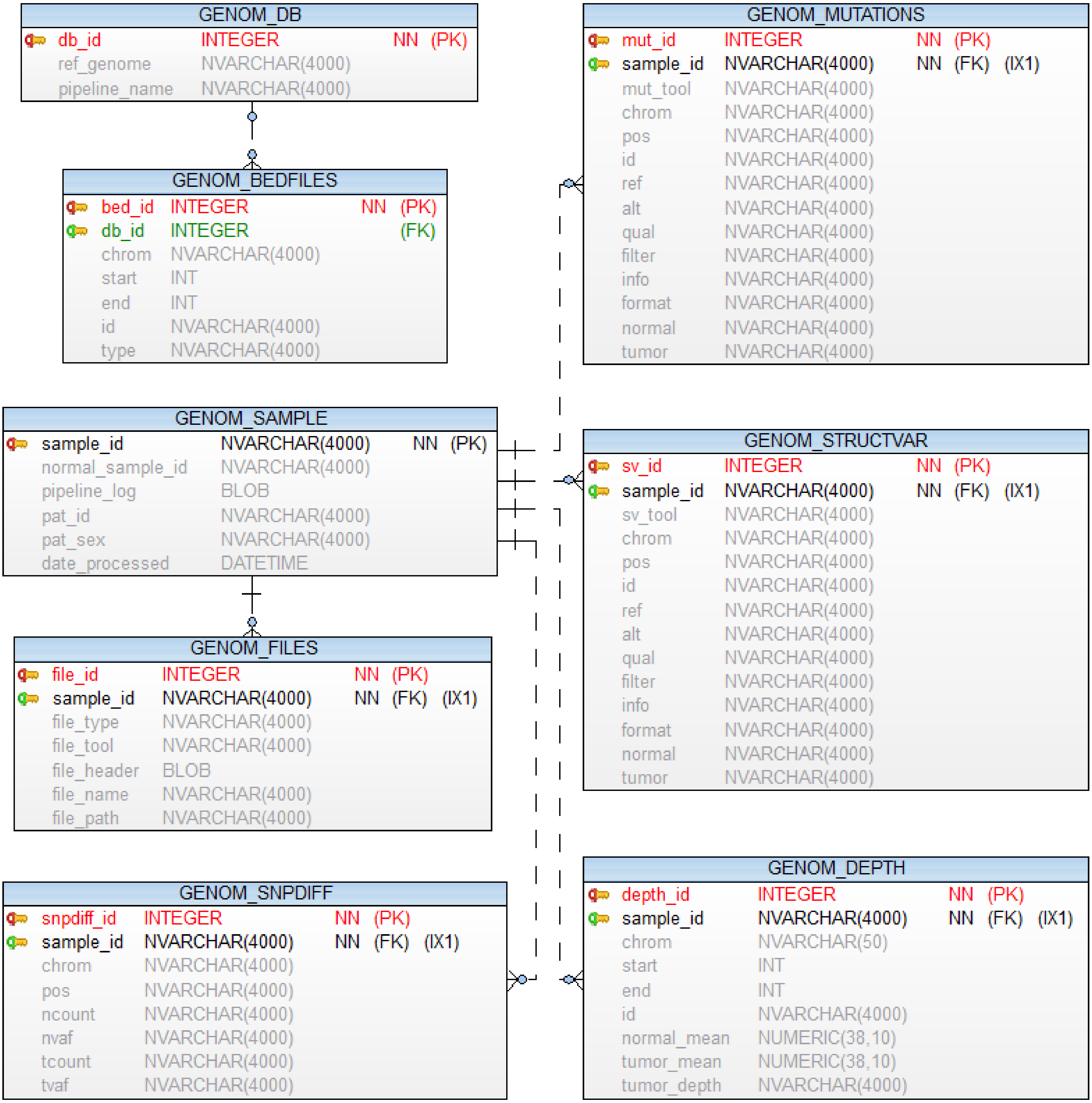
GENOM_DB
db_id INTEGER NN (PK)
ref_genome NVARCHAR(4000)
pipeline_name NVARCHAR(4000)
GENOM_BEDFILES
bed_id INTEGER NN (PK)
db_id INTEGER (FK)
chrom NVARCHAR(4000)
start INT
end INT
id NVARCHAR(4000)
type NVARCHAR(4000)
GENOM_SAMPLE
sample_id NVARCHAR(4000) NN (PK)
normal_sample_id NVARCHAR(4000)
pipeline_log BLOB
pat_id NVARCHAR(4000)
pat_sex NVARCHAR(4000)
date_processed DATETIME
GENOM_FILES
file_id INTEGER NN (PK)
sample_id NVARCHAR(4000) NN (FK) (IX1)
file_type NVARCHAR(4000)
file_tool NVARCHAR(4000)
file_header BLOB
file_name NVARCHAR(4000)
file_path NVARCHAR(4000)
GENOM_SNPDIFF
snpdiff_id INTEGER NN (PK)
sample_id NVARCHAR(4000) NN (FK) (IX1)
chrom NVARCHAR(4000)
pos NVARCHAR(4000)
ncount NVARCHAR(4000)
nvaf NVARCHAR(4000)
tcount NVARCHAR(4000)
tvaf NVARCHAR(4000)
GENOM_MUTATIONS
mut_id INTEGER NN (PK)
sample_id NVARCHAR(4000) NN (FK) (IX1)
mut_tool NVARCHAR(4000)
chrom NVARCHAR(4000)
pos NVARCHAR(4000)
id NVARCHAR(4000)
ref NVARCHAR(4000)
alt NVARCHAR(4000)
qual NVARCHAR(4000)
filter NVARCHAR(4000)
info NVARCHAR(4000)
format NVARCHAR(4000)
normal NVARCHAR(4000)
tumor NVARCHAR(4000)
GENOM_STRUCTVAR
sv_id INTEGER NN (PK)
sample_id NVARCHAR(4000) NN (FK) (IX1)
sv_tool NVARCHAR(4000)
chrom NVARCHAR(4000)
pos NVARCHAR(4000)
id NVARCHAR(4000)
ref NVARCHAR(4000)
alt NVARCHAR(4000)
qual NVARCHAR(4000)
filter NVARCHAR(4000)
info NVARCHAR(4000)
format NVARCHAR(4000)
normal NVARCHAR(4000)
tumor NVARCHAR(4000)
GENOM_DEPTH
depth_id INTEGER NN (PK)
sample_id NVARCHAR(4000) NN (FK) (IX1)
chrom NVARCHAR(50)
start INT
end INT
id NVARCHAR(4000)
normal_mean NUMERIC(38,10)
tumor_mean NUMERIC(38,10)
tumor_depth NVARCHAR(4000)
