# Supplementary figures and images for: TarPan: an easily adaptable targeted sequencing panel viewer for research and clinical use
Source: BMC Bioinformatics. 2020 Apr 15;21:144. doi: 10.1186/s12859-020-3477-y (PMC7158102; doi:10.1186/s12859-020-3477-y)

# Chromosome 1

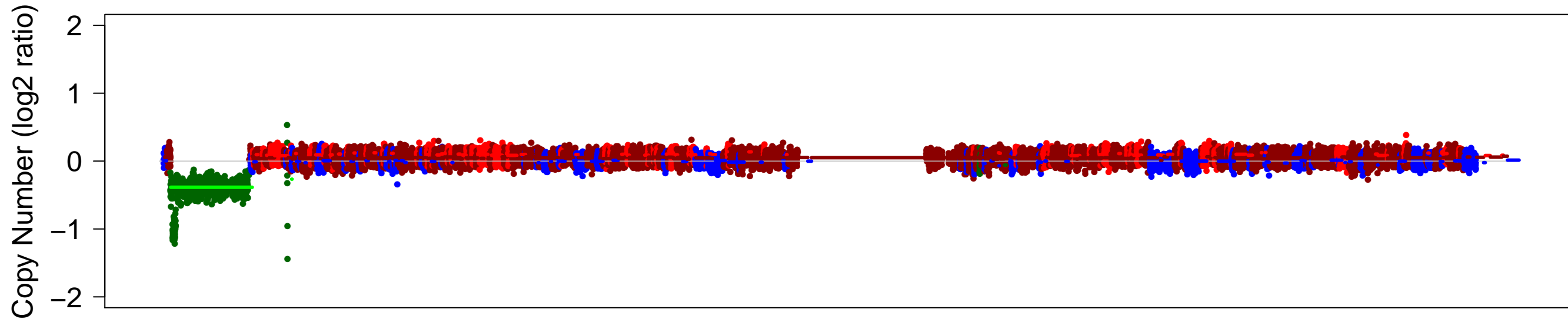

Supplement: Supplementary file 2 — Additional file 2. [file 12859_2020_3477_MOESM2_ESM.pdf]

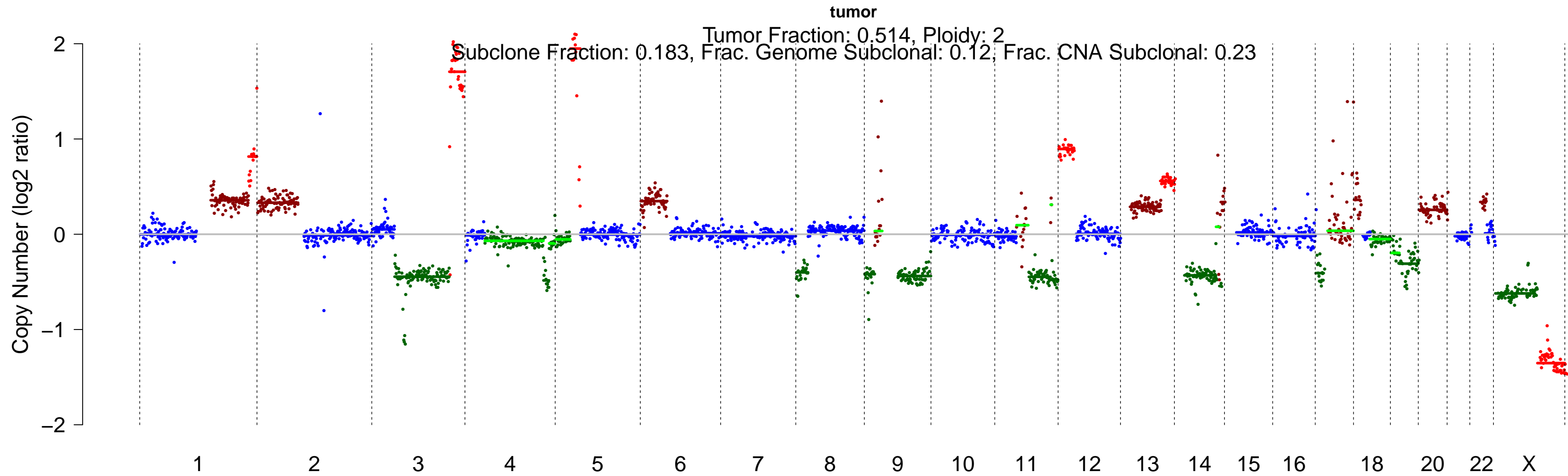

Supplement: Supplementary file 3 — Additional file 3. [file 12859_2020_3477_MOESM3_ESM.pdf]
